# Cancer patients’ perspective on shared decision-making and decision aids in oncology

**DOI:** 10.1007/s00432-021-03579-6

**Published:** 2021-03-07

**Authors:** Lena Josfeld, Christian Keinki, Carolina Pammer, Bijan Zomorodbakhsch, Jutta Hübner

**Affiliations:** 1 Klinik für Innere Medizin II Hämatologie und Internistische Onkologie, Universitätsklinikum Jena, Am Klinikum 1, 07747 Jena, Germany; 2 üBAG/MVZ Onkologische Kooperation Harz GbR, Kösliner Str. 14, 38642 Goslar, Germany

**Keywords:** Shared decision-making, Patient decision aids, Patient satisfaction, Patient-clinician communication, Oncological patients, Complementary and alternative medicine

## Abstract

**Purpose:**

Shared Decision-Making (SDM) enhances patients’ satisfaction with a decision, which in turn increases compliance with and adherence to cancer treatment. SDM requires a good patient-clinician relationship and communication, patients need information matching their individual needs, and clinicians need support on how to best involve the individual patient in the decision-making process. This survey assessed oncological patients’ information needs and satisfaction, their preferred information in patient decision aids (PDAs), and their preferred way of making decisions regarding their treatment.

**Methods:**

Questionnaires were distributed among attendees of a lecture program on complementary and alternative medicine in oncology of which 220 oncological patients participated.

**Results:**

Participants reported a generally high need for information—correlating with level of education—but also felt overwhelmed by the amount. The latter proved particularly important during consultation. Use of PDAs increased satisfaction with given information but occurred in less than a third of the cases. Most requested contents for PDAs were pros and cons of treatment options and lists of questions to ask. The vast majority of patients preferred SDM to deciding alone. None wanted their physician to decide for them.

**Conclusions:**

There is a high demand for SDM but a lack of conclusive evidence on the specific information needs of different types of patients. Conversation between patients and clinicians needs encouragement and support. PDAs are designed for this purpose and have the potential to increase patient satisfaction. Their scarce use in consultations calls for easier access to and better information on PDAs for clinicians.

## Introduction

The exceedingly complex considerations when making decisions about cancer treatment are a challenge not only for patients to comprehend, but also for physicians to present and explain to their patients in the limited amount of time they usually have during a consultation. There is often too much potentially important information to take into account. In recent decades, patients’ autonomy and participation in medical decision-making processes has been encouraged and become a widely accepted goal. But in order for them to participate they require sufficient information.

Previous research shows a generally high demand in patients for information concerning their disease and treatment (Gaston and Mitchell 2005). A systematic review encompassing studies between 2000 and 2012 (Pieper et al. 2015) suggests a higher demand from younger patients, female patients, patients who have not been diagnosed for very long, and those who are in rather bad health or exhibit more anxious and depressive symptoms. Demand is less in patients who show higher satisfaction with their physician, trust the nurses, receive more care, and who experience more empathy from their physicians.

Beside general information needs, Butow et al. found already in 2004 an overall preference for shared decision-making (SDM) in patients. However, implementation of SDM in clinical practice has hardly improved over time (Vogel et al. 2008; Wiener et al., 2018), despite evidence to its benefits. Patients tend to be more satisfied with decisions about their treatment when they feel they have been involved in the process, regardless of whether they explicitly preferred SDM before the actual consultation or not (Brown et al. 2012). There are, however, differences in patients’ decision-making preferences, which may have to be taken into account when trying to successfully implement SDM: The younger patients are, the more they want to be involved (Butzlaff et al. 2003). Gender (female) and education level (higher) has also been associated with preferring SDM (Gaston and Mitchell 2005). On the part of physicians, empathy during a consultation appears to be positively correlated to SDM and negatively correlated to patients’ regret after a decision has been made (Nicolaia 2016). Patients preferring share decision-making (as opposed to decisions made solely by the physician or themselves) have been found to have high but not excessive levels of trust in their physician (Kraetschmer et al. 2004).

When selecting and presenting patient information, there are ethical issues to consider but also central methodical aspects to enable patients to actively participate in decision-making while avoiding potential harm (Middel 2011). An increasingly popular approach to help patients take an active part in the decision-making process is the use of patient decision aids (PDAs). They cater to patients’ information needs, explain the decision that has to be made, and endorse the patients’ active role in it. A recent study found that cancer patients primarily require information on treatment experience, post-treatment quality of life, and the impact of side effects, while clinicians are more focussed on clinical outcome in their consultations (Ankolekar et al. 2019). Another potential problem is the evidence of decisive differences between what patients and physicians perceive as actual decisions during a consultation (Hargraves et al. 2016). Both these findings emphasize the previously proclaimed necessity for patients and clinicians to actively communicate and discuss important issues, to truly share in the decision-making process (Feldman-Stewart et al. 2013). Patient decision aids attempt to close these gaps by providing all potentially relevant information in a patient-centred way to facilitate communication for both sides.

Research findings on the benefits of PDAs are heterogeneous: They generally enhance knowledge and the feeling of being well informed, but may or may not have an influence on decision conflicts and patient satisfaction, anxiety or depression (Krassuki et al. 2019; McAlpine et al. 2018; Spiegle et al. 2013; Scalia et al. 2019; Stacey et al. 2017; Vodermaier et al. 2009). Specific attributes of decision aid content and format have not been found to mediate their effectiveness, as long as they meet sufficient standards (Trikalinos et al. 2014) as described by the International Standard for Patient Decision Aids (IPDAS) (Elwyn et al. 2006; Joseph-Williams et al. 2014). Handing out patient information material shortly before medical consultation does not necessarily encourage SDM during consultation (Butow et al. 2004). Evidence suggests it is important that the physician is the one giving the information and actively involving the patient in the decision-making process to enhance trust and avoid mistrust (Nannenga et al. 2009).

To summarize, Shared decision-making enhances patients’ satisfaction with a decision and is a relevant factor for compliance with and adherence to cancer treatment. To support this kind of decision-making process, patients need an amount of information which remains manageable and is not overwhelming but at the same time addresses what is important to them. They also need a good working relationship with their physician based on trust. This may be enhanced by empathy as well as the physician being the one giving the patients access to the information they require. Physicians need easy-to-access guidelines on how to best involve the individual patient in the decision-making process. The question remains how those heterogenous findings on PDAs may be explained and how they can be more successfully implemented.

This study assumes that the appropriate amount and type of information in PDAs may vary depending on patient and case characteristics—particularly what kind of decision patients are faced with at which point of their treatment process, their educational background, possibly age and gender as well. The crucial point may be the proper fit between the individual patient, their current situation and preferred way of decision-making on one hand, and the type of decision aid and information they receive on the other.

Specifically, this study assesses (1) how well-informed oncological patients feel in general about decisions concerning their treatment, (2) what kind of information patients with different characteristics would like to receive in a decision aid, and (3) what their preferences are regarding the decision-making process. The findings may contribute to a better understanding of how to match fitting information to different patients to improve the benefits of decision aids and facilitate SDM.

## Methods

### Participants

All participants were recruited from a lecture program on complementary and alternative medicine (CAM) which was held by the working group Prevention and Integrative Oncology of the German Cancer Society in 11 different cities all over Germany, running from January through December 2017. The lectures were gratuitous and open to everyone interested. They addressed cancer patients and their caregivers. All lectures were held by a specially trained oncologist in non-expert language and provided evidence-based information.

### Questionnaire

The questionnaire was a standardized set of questions consisting of five sections:

1. Demographic data and tumour specific data
2. Decisions regarding therapy (e.g., therapy against side effects, accompanying measures like nutrition, exercise, discontinuation of therapy, changes in therapy)
3. Satisfaction with information given by the physician, need for more or less information (before, after or by the time of the doctor-patient discussion)
4. Information required in a decision aid (e.g., detailed information in a text, a short summary, a comparison of pros and cons, graphic presentation, question to ask the physician, experiences and reports of other patients, free spaces for own questions, automatic summary)
5. Demand to participate in decision on therapy (decision by patient alone, shared with the physician or by the physician alone)

The questionnaire has been developed by members of the working group Prevention and Integrative Oncology of the German Cancer Society. It contained closed questions that could be answered with a single answer (e.g., “yes”, “no”, “I don’t know”) as well as questions with rating on a Likert scale (e.g., the question regarding the satisfaction with the information given by the physician; ranging from “1 not at all satisfied” to “5 very satisfied”), multiple selection questions (e.g., “Which decision did you have to make during your disease”, “What would be important for you in a decision aid?”) and open answers.

## Statistics

IBM SPSS Statistics 25 was used for data collection and analysis. Associations between information needs and gender, age and education were tested using chi-square tests; correlations between satisfaction with given information and decision aids were tested via Welch-test; influential parameters on decision-making preferences were searched for via correlations and multinomial regression analyses; p < 0.05 was considered significant.

## Results

### Demographic data

Two hundred and twenty patients answered a questionnaire during a course of lectures taking place in different cities in Germany. The majority were patients currently under treatment (59.1%), 90 (40.9%) had already completed their treatment. Age ranged between 28 and 86 years, with a mean of 76.2 years and median of 65 years. The largest group of patients in the survey (33.6%) was diagnosed with breast cancer; almost half of the participants (48.1%) reported high education levels. Ninety-two (34.3%) of the interviewed people were male, 154 (75.7%) were female. Detailed characteristics of the study group are shown in Table 1.

**Table 1:** Demographic data of participants

	N (% of valid answers)
Status	
Currently under treatment	130 (59.1)
After treatment	90 (40.9)
Type of cancer	
Breast cancer	87 (40.5)
Gastrointestinal cancer	29 (13.5)
Prostate cancer	29 (13.5)
Leukaemia/Lymphoma	25 (11.6)
Others	45 (20.9)
Age	
< = 40	9 (4.1)
41–60	80 (36.5)
61–70	71 (32.4)
> 70	59 (26.9)
Gender	
Female	130 (63.1)
Male	76 (36.9)
Education level	
High	104 (54.7)
Intermediate	51 (26.8)
Low	35 (18.4)

### Satisfaction with given information

**Fig. 1:**
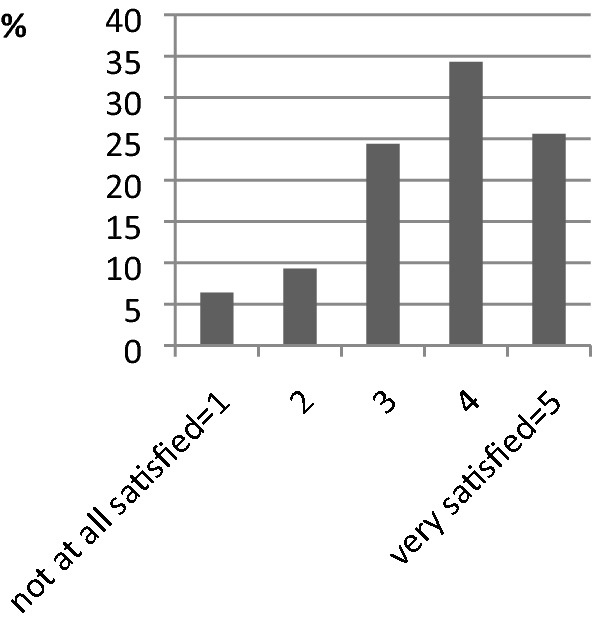
Satisfaction with information given by physician (N=172)

Regarding patients’ satisfaction with information they had been given, the mean of all valid answers (*N* = 172 of 220) on a five-point scale between 1 = ‘not satisfied at all’ and 5 = ‘very satisfied’ was 3.63 (SD = 1.15) (Fig. 1).

### Information needs

**Fig. 2:**
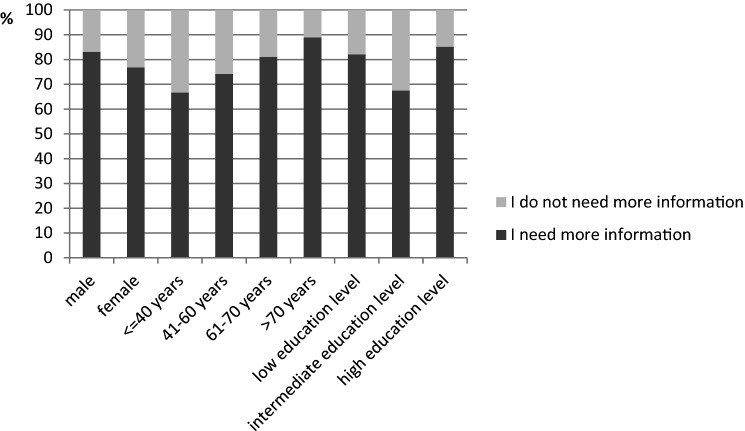
Information needs with respect to gender, age, and level of education

Overall, the majority of participants (60.5%, *N* = 133 of 167 answering this question) required more information than they had received so far. The Chi^2^-test revealed no difference in the information needs regarding age (*χ*^2^(3) = 4.256, *p* = 0.235) or gender (*χ*^2^(1) = 0.852, *p* = 0.356). We found merely a tendency towards a correlation between higher level of education and higher need for information, with a small effect, but this was not significant (*χ*^2^(2) = 5.319, *p* = 0.07, Cramer’s *V* = 0.189). The differences are illustrated in Fig. 2.

Chi-square tests found no relations between information needs and type of cancer or type of decision patients were facing.

Patients reported both receiving too little and too much information. Looking at the point in the decision-making process at which the amount of information appears most crucial, participants indicated that it is during the consultation with the physician (as opposed to before or after) when most of them felt either overwhelmed or underinformed (Table 2).

**Table 2:** Amount of information by point in time (Frequencies in %)

	Too little information	Too much information
Before consultation	19.5	3.6
During consultation	31.8	10.9
After consultation	11.4	5.9

### Decision-making preferences

#### Decisions participants faced during the trajectory of their disease

**Fig. 3:**
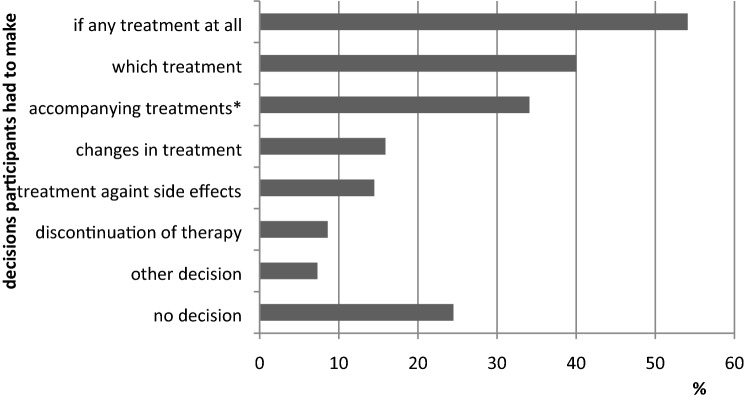
Decisions patients reported they had to make regarding their therapy (N=220). *i.e., physical activity, nutrition, naturopathy

Most patients (*N* = 119 of 220; 54.1%) reported they had to decide whether they wanted to receive a therapy, followed by the decision about the type of treatment (*N* = 88; 40%). Almost a quarter (54; 24.5%) reported they did not have to make any decision at all (Fig. 3).

#### Decision aids and required elements

**Fig. 4:**
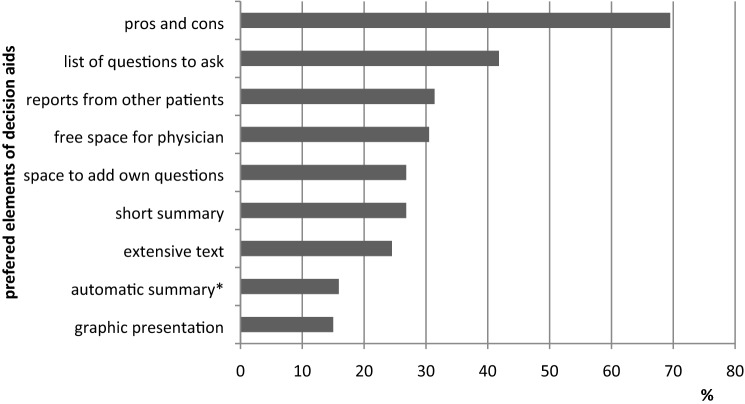
Information patients would like to have in a decision aid (N=220). *i.e., interactive in an online portal or an app

Most patients (*N* = 103 of 170 valid answers; 60.6%) reported they had not received any decision aid, only 50 (29.4%) did, while seventeen (10%) were not sure. The questionnaire also assessed which type of information patients would like to receive in a decision aid. The most important elements of a decision aid were judged to be a comparison of pros and cons (*N* = 153; 69.5%) and a list of questions to ask the physician (*N* = 92; 41.8%). Experiences or reports of other patients and space for the physician to write down individual information for the patient were stated as important content as well (Fig. 4).

Neither type of cancer nor the kind of decision patients had to face showed an influence on what kind of information patients would prefer in a decision aid. Only a list of questions to ask was chosen significantly more often by patients with low or intermediate levels of education than patients with a high education level (*χ*^2^(2) = 6.752, *p* = 0.034, Cramer’s *V* = 0.189). Inclusion of an extensive text showed a tendency towards being picked more often by patients with a high education level, but this was not significant (*χ*^2^(2) = 5.093, *p* = 0.078, Cramer’s *V* = 0.164).

Patients who received a decision aid were significantly more satisfied with information given by their physician (*T*(134.72) = 3.356, *p* = 0.001). The effect was approaching medium size (Cohen’s d = 0.45).

### Shared-decision-making

Fifteen of the participants (8.6% of 175 answers) wanted to make therapeutic decisions alone. The vast majority (*N* = 160, 91.4%) preferred to decide together with their physician. None wanted the physician to decide alone. Sixty-seven participants (30.45% of the total sample) did not answer this question. Due to low variance among answers to this question, the results of the following analyses of correlations as well as multinomial regression analyses regarding decision-making preferences cannot be considered conclusive. Neither analysis could identify significant predictors for decision-making preferences among age, gender, level of education, type of cancer, type of decision, and satisfaction with given information.

## Discussion and conclusion

### Discussion

This survey found a high demand among cancer patients for more information when it comes to decisions about their treatment, regardless of their socio-demographic background, type of cancer or current status of treatment. Overall, given information has been leaning towards satisfactory, but many patients reported they felt overwhelmed or underinformed at different points during the process. Especially during a consultation—more so than before or after it—the amount of information given to a patient appears to be crucial and has to match their individual needs.

Patient decision aids had not been used often to give information. There may be a multitude of reasons ranging from lack of awareness on the part of the physician, uncertainty about their quality and how to assess it, to ambiguous evidence for their effects. As Wiener et al. (2018) found, clinicians have reported difficulties in accessing PDAs. Where they are being used, this study found they significantly increase patients’ satisfaction with the information given by their physician.

According to growing scientific findings, differences in content and format of decision aids seem not to influence their overall effectiveness (Wiener et al. 2018). However, besides the IPDAS criteria, certain elements have previously been found particularly helpful from patients’ point of view: a summary of the consultation (Pitkethly et al. 2008), quantitative information on risks (Trevena et al. 2013), and personalised information with regard to individual prognosis (Mühlbauer et al. 2019), to type of cancer and to which information matters most to patients (Jones et al. 2006). This study finds that patients’ opinions vary regarding the type of required information and the way it should best be presented. Generally, what participants have been most interested in were the pros and cons of the different options, a list of questions to ask their physician, reports from other patients and the possibility for the physician to add personalised information.

The various factors accounting for differences in the requested amount and type of information appear difficult to identify. Age and gender showed no effect in this group, neither did type of cancer and type of decision, which may arise from a generally high interest in information among the study’s participants due to the lecture setting. More differences in the preferred type and format of information could have been expected to depend on education level, but the only difference found in this regard concerned the list of questions patients could ask their physician. This was requested more often by people with low to intermediate levels of education. A tendency towards the opposite was discovered with regard to an extensive information text, as well as a tendency for patients with higher education to generally require more information.

Patients in this survey unanimously want to participate in decision processes about their treatment, and the vast majority prefer to decide together with their physician instead of alone. This result is surprising as previous research found a considerable percentage of patients preferring their physicians to make the decisions for them (Efficace et al. 2014; Mazur et al. 2005; Mazur and Hickam 2005) correlating with age and numerical reasoning (Galesic and Garcia-Retamero 2011). The present study’s result may originate in the particular characteristics of its participants, who were all interested in lectures about cancer treatment in general and use of complementary and alternative medicine in particular. This supports previous indications that in consultations on CAM, SDM plays a more central role than in conventional medicine (Berger et al. 2012).

### Limitations

Participants of this survey were recruited from a fairly selective group of people interested in CAM and willing to attend lectures held by a clinical expert, which suggests a higher personal involvement in the treatment of their illness. This may account for the overwhelming majority of patients preferring SDM, as well as the complete absence of patients preferring their physician to make decisions for them.

The differences between shared decision-making (Eddy 1990; Elwyn et al. 2010) and informed consent (Beauchamo and Childress 2009) are often blurred in practice, sometimes in research as well, but especially for laypeople (Kunneman and Montori 2017; Shahu et al. 2017). Though we asked for ‘making decisions together with the physician’ it remains unclear, whether participants’ answers referred to true SDM, or whether they simply indicated they did not want a decision to be made over their heads and want to receive adequate information (which would qualify as informed consent). Another possibility to consider is a socially desirable answer. It may be a widely spread expectation by now, that patients actively participate in the decision-making process. Accordingly, participants of this study may have conformed to this expectation in their answers.

### Conclusion

The study revealed a high demand for SDM among cancer patients, particularly those interested in CAM. The latter may be due to those patients’ intrinsic values or to a heightened awareness of SDM in this field in contrast to conventional medicine. However, implementation of SDM in clinical practice is still problematic and somewhat rare. During the limited time available, physicians face the challenge of assessing the specific information needs of individual patients to enable them to partake in the decision-making process. This study shows that these needs vary greatly and make it difficult to build heuristics for this challenge.

There seem to be two paths to take to facilitate SDM: On one hand, further research should focus on matching the individual patient in their current situation to the type and amount of information they receive. Based on these results, specialised and simple tool-kits may be developed which would enable oncologists to quickly decide how to present which kind of information and how to facilitate SDM processes, depending on what the specific patient requires in their specific situation. On the other hand, patients and physicians should be supported in finding ways to communicate more effectively with each other. Where scientific research cannot yet adequately summarize patients’ needs, patients may be educated to express them directly, physicians may be educated on how to best ascertain them.

With SDM as the gold standard of clinical decisions in oncology, PDAs are a promising if underused tool. They are designed to aid patient-clinician communication and can be adapted for different types of patients with different needs. Already, they reportedly increase patients’ satisfaction with received information. To include question prompt lists in a PDA may help encourage conversation between clinicians and patients with lower education levels in particular.

The comparatively rare use of PDAs in everyday practice needs to be addressed not only in scientific but public discussion with clinicians as well. If future research can shed more light on which type of PDA best matches the needs of particular groups of patients, clinicians may be encouraged to use them more. An inclusion into clinical guidelines might also be conceivable. The question remains how to reach and involve patients who want to hand over the entire responsibility for their health and treatment to clinicians. This will be another central task for future research to approach.

## Data Availability

The datasets generated and analysed during the current study are available from the corresponding author on reasonable request.
